# Evolutionary, computational, and biochemical studies of the salicylaldehyde dehydrogenases in the naphthalene degradation pathway

**DOI:** 10.1038/srep43489

**Published:** 2017-02-24

**Authors:** Baolei Jia, Xiaomeng Jia, Kyung Hyun Kim, Zhong Ji Pu, Myung-Suk Kang, Che Ok Jeon

**Affiliations:** 1 School of Bioengineering, Qilu University of Technology, Jinan 250353, China; 2 Department of Life Science, Chung-Ang University, Seoul 06974, Republic of Korea; 3 School of Life Science and Biotechnology, Dalian University of Technology, Dalian 116024, China; 4 Microorganism Resources Division, National Institute of Biological Resources, Incheon 22689, Republic of Korea

## Abstract

Salicylaldehyde (SAL) dehydrogenase (SALD) is responsible for the oxidation of SAL to salicylate using nicotinamide adenine dinucleotide (NAD^+^) as a cofactor in the naphthalene degradation pathway. We report the use of a protein sequence similarity network to make functional inferences about SALDs. Network and phylogenetic analyses indicated that SALDs and the homologues are present in bacteria and fungi. The key residues in SALDs were analyzed by evolutionary methods and a molecular simulation analysis. The results showed that the catalytic residue is most highly conserved, followed by the residues binding NAD^+^ and then the residues binding SAL. A molecular simulation analysis demonstrated the binding energies of the amino acids to NAD^+^ and/or SAL and showed that a conformational change is induced by binding. A SALD from *Alteromonas naphthalenivorans* (SALDan) that undergoes trimeric oligomerization was characterized enzymatically. The results showed that SALDan could catalyze the oxidation of a variety of aromatic aldehydes. Site-directed mutagenesis of selected residues binding NAD^+^ and/or SAL affected the enzyme’s catalytic efficiency, but did not eliminate catalysis. Finally, the relationships among the evolution, catalytic mechanism, and functions of SALD are discussed. Taken together, this study provides an expanded understanding of the evolution, functions, and catalytic mechanism of SALD.

Naphthalene (C_10_H_8_; CAS number 91-20-3), which is the most abundant polycyclic aromatic hydrocarbon (PAH), is a contaminant that is found environmentally as a constituent of coal tar, crude oil, and cigarette smoke^1^. Naphthalene and its substituted derivatives are also used in chemical manufacturing as a chemical intermediate for many commercial products ranging from pesticides to plastics. Humans are exposed to naphthalene through a wide range of mechanisms, resulting in the production of reactive metabolites that deplete glutathione and result in oxidative stress^2^. Based on its abundance and toxicity, naphthalene has been identified as a priority pollutant and a possible human carcinogen by the Environmental Protection Agency of the USA^3^. As the simplest PAH, naphthalene has been used as a model compound for studies on the metabolism of PAHs by microorganisms^4^.

Chemical, physical, and biological methods have been used for naphthalene remediation^5^. Above all, microbial biodegradation methods have been favored because of their environmental-friendliness, effectiveness, and low costs^6^. Bacterial strains isolated from contaminated soil or sediments such as *Pseudomonas* spp., *Bacillus* spp.^7^, *Burkholderia* spp., *Comamonas* spp., and *Rhodococcus* sp.^6^ are some of the best-studied naphthalene-degrading bacteria. Our previous work demonstrated that *Alteromonas naphthalenivorans* is a key biodegrader of PAH in crude oil-contaminated coastal sediment by two years of monitoring^8^. PAH bi,odegradation using filamentous fungi (including white rot fungi) such as *Phanerochaete chrysosporium, Pleurotus ostreatus*, and *Trametes versicolor* has been reported^9^,^10^. Some *Ascomycota* fungi such as *Fusarium* sp. and *Aspergillus* sp. have also been reported to degrade naphthalene^11^. The majority of reported naphthalene degradation pathways in bacteria are aerobic and can be divided into two stages: the upper pathway transforms naphthalene to salicylate and the lower pathway converts salicylate to tricarboxylic acid cycle intermediates through meta-cleavage pathway enzymes^12^. The fungi metabolize naphthalene with the enzymes lignin peroxidase, manganese peroxidase, laccase, cytochrome P450, and epoxide hydrolase^13^.

During naphthalene degradation in bacteria, salicylaldehyde (SAL) dehydrogenase (EC 1.2.1.65, denoted as SALD) catalyzes the oxidation of SAL to salicylate using NAD^+^ as a cofactor. SALD is considered to be the last enzyme in the upper catabolic pathway and it plays an important role in connecting the upper pathway to the lower catabolic pathway, which leads to the production of tricarboxylic acid cycle intermediates^12^. Two genes encoding SALD were discovered in *Pseudomonas putida* ND6, namely *NahV* and *NagF*, which showed a 72% identity to each other in their conserved regions. The corresponding enzymes showed different but overlapping properties, thus ensuring that single gene mutants could survive in naphthalene-containing environments^14^,^15^. The SALD from *Pseudomonas* sp. strain C6. was found to be a functional homotrimer and showed a broad substrate specificity^16^. The crystal structure of the SALD from *P. putida* G7 (SALDpp) was determined and showed α/β folding with three domains, namely the oligomerization, cofactor-binding, and catalytic domains. The SAL was buried in a deep pocket in the structure where the catalytic Cys284 and Glu250 residues were located. The cysteine residue was able to attack the carbonyl carbon of the substrate and the glutamic acid residue functioned as a general base. In addition, the residues Arg157, Gly150, and Trp96 were found to play an important role in determining the specificity of the enzyme for aromatic and aliphatic aldehyde dehydrogenases^17^,^18^.

SALD belongs to the aldehyde dehydrogenase (EC 1.2.1.3) superfamily, the members of which are responsible for the oxidation of a wide variety of aliphatic and aromatic aldehydes to carboxylic acids using nicotinamide adenine dinucleotide (NAD^+^) or nicotinamide adenine dinucleotide phosphate (NADP^+^) as a coenzyme. The overall reaction catalyzed by the aldehyde dehydrogenases is: RCHO + NAD^+^ + H_2_O → RCOOH + NADH + H^+^. Our previous work demonstrated that SALD from *A. naphthalenivorans* (SALDan) was specifically up-regulated in response to naphthalene^19^. To gain insights into the function, evolution, and catalytic mechanism of SALD, we performed a comprehensive analysis using a sequence similarity network (SSN), a phylogenetic tree, molecular dynamics (MD), kinetics, and mutation analysis using SALDan (Uniprot AC: F5Z5S7) as a template. SALDan showed 63% identity to SALDpp (Uniprot AC: Q1XGL7). We found that SALD is present in both bacteria and fungi. The catalytic cysteine and the amino acids binding NAD^+^ have been highly conserved during evolution. SALDan can bind NAD^+^ strongly and SAL binding can decrease the binding energy to NAD^+^. We further purified the wild-type and mutant SALDan proteins. Biochemical assays of the enzymes supported the evolutionary and MD analysis results.

## Results

### Distribution and evolution of SALD

**Figure 1:**
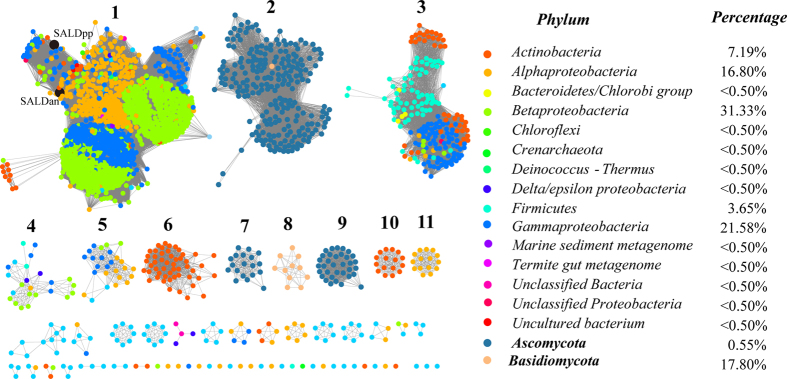
Taxonomic distribution of SALDs. The proteins listed in Supplementary Dataset 1 were used to generate the network using an e-value of 10^−150^ (>60% sequence identity). Each node represents one protein. Edges are shown with BLASTP e-values below the indicated cutoff. A cluster was sequentially labeled if there were more than 10 nodes in that cluster. Nodes from the same taxonomic groups in the global network are the same color. The nodes representing SALDan and SALDpp are highlighted by a black coloration and large size. The colors corresponding to each phylum and protein percentage in each phylum are listed on the right.

To determine the distribution of SALD in the biosphere, the protein sequences were acquired by searching the UniProt database^20^ using SALDan sequence as a query with an e-value threshold of 10^−80^ (>40% sequence identity). This threshold was chosen because several studies have shown that sequences that share >40% identity are very likely to share functional similarity, as judged by Enzyme Commission numbers^21^. In total, 2039 proteins were obtained and listed in Supplementary Dataset 1. To further clarify the distribution of these proteins and their relationships, a network for 2039 protein sequences was constructed using the Enzyme Function Initiative-Enzyme Similarity Tool^22^ with an e-value threshold of 10^−150^ (>60% sequence identity was the cutoff for an edge between nodes [i.e., proteins]). The proteins were mainly separated into 11 groups and each protein was painted according to its taxonomic classification (Fig. 1). Further analysis of the proteins from NCBI metagenomics protein database (env_nr) showed that the homologues of SALD from environmental samples were able to be classified into group 1, 5, and 11 (Supplementary Fig. 1). Members of the enzymes were found in the domain “bacteria” and kingdom “fungi”. In bacteria, at the class level, the proteins were mainly distributed in *Actinobacteria* (7.19%), *Alphaproteobacteria* (16.80%), *Betaproteobacteria* (31.33%), *Firmicutes* (3.65%), and *Gammaproteobacteria* (21.58%). In fungi, at the phylum level, the prevalence of *SALD* genes was 0.55% in *Ascomycota* and 17.80% in *Basidiomycota*.

**Figure 2:**
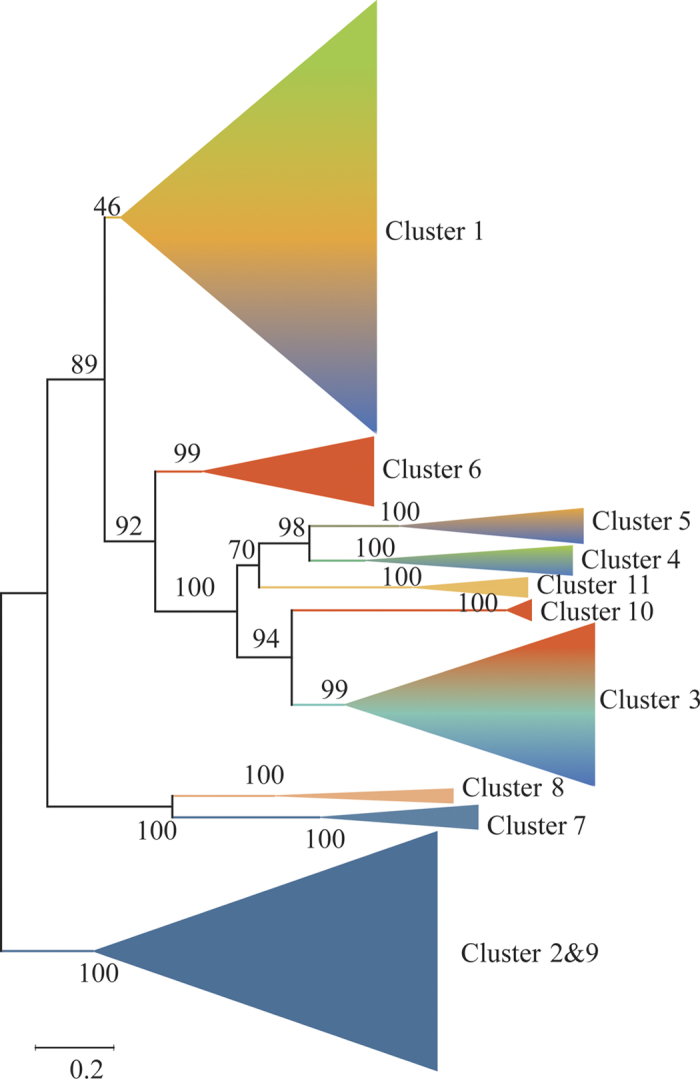
Maximum likelihood phylogenetic tree for 2039 proteins from bacteria and fungi generated using MEGA. The tree with the highest log likelihood (−325820.9727) is shown. The percentage of replicate trees in which the associated taxa clustered together in the bootstrap test (1000 replicates) is shown next to the branches. The color of the branch corresponds to the color of the cluster in Fig. 1.

To provide a more detailed view of the evolutionary relationships across the groups, we performed a phylogenetic analysis using the proteins in the clusters assigned based on sequence comparisons (Fig. 2). Proteins from the same cluster always clustered together and were well-separated in the phylogenetic tree, except that clusters 2 and 9 from *Ascomycota* were clustered in the same branch. The separation of these groups had a high level of bootstrap support in the phylogenetic tree. Meanwhile, the proteins from bacteria were gathered in a clade with a high level of bootstrap support. The proteins from fungi formed separate branches in the phylogenetic tree. Cluster 7 from *Ascomycota* and cluster 8 from *Basidiomycota* were much closer to the proteins of bacteria and clusters 2 and 9 formed a distinct branch.

### Conservation and coevolution of amino acids in SALD sequences

**Figure 3:**
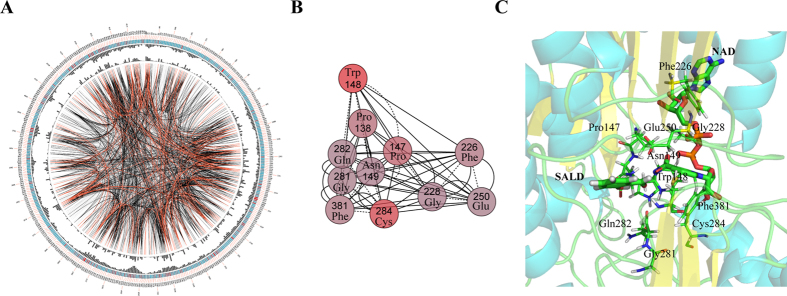
Conserved and coevolved amino acid residues in SALDs represented using SALDan as a reference sequence. (**A**) Network analysis of coevolving residues. The circular network shows the connectivity of coevolving residues. The labels in the first circle indicate the alignment position and the amino acid code of SALDan. The colored square boxes of the second circle indicate the multiple sequence alignment position conservation (highly conserved positions are colored red, whereas less conserved ones are colored blue). The third and fourth circles show the proximity mutual information (MI) and cumulative MI (cMI) values as histograms, facing inward and outward, respectively. In the center of the circle, the edges that connect pairs of positions represent a significant MI value (>6.5), highlighted using red lines to show a higher MI score (top 5%), black ones for a moderately high MI score (between 70% and 95%), and gray ones for a lower MI score (the remaining 70%) as defined by MISTIC. (**B**) The network of cMI with a high conservation value. Nodes represent the top 11 most-conserved amino acid residues (labeled with position and code) and nodes are colored by conservation from red (higher) to blue (lower). The length of the edges is inversely proportional to MI value (the closest nodes have higher MI). (**C**) A cartoon diagram of SALDan with the top coevolved and conserved amino acid residues.

To examine the conservation and coevolution of primary sequences of SALD, we first determined consensus sites based on multiple sequence alignments (MSA) of 2039 sequences using SALDan as the reference sequence to display MSA and the conservation of the residues (Fig. 3). The most highly conserved amino acid was found to be Cys284 (Fig. 3A,B), which functions as the active site nucleophile in the dehydrogenase reaction. The other conserved amino acids were always hydrophobic, including Pro147, Asn149, Phe226, Gly228, Gly281, Gln282, and Phe381. Glu250, which may interact with the aldehyde substrate, is also in the list. We further investigated the coevolution of SALD amino acids using mutual information (MI)^23^ (Fig. 3A,B). If two residues share a high MI score, they are most likely coevolving, meaning that to maintain a given enzymatic function, a mutation of one residue is linked to a specific compensatory mutation of the other residue^24^,^25^. The MI network for 2039 SALD members revealed that higher MI values (the top 10% of MI values) were evenly distributed across all amino acid positions from the N-terminus to the C-terminus (Fig. 3A). The 11 most conserved residues were chosen for further analysis. These conserved residues formed a connected network, indicating that these residues also shared a significant MI value (Fig. 3B). The strong correspondence observed between the conserved residues and the coevolving residue positions is consistent with previous studies^26^,^27^. Mapping the top coevolving and conserved residues onto the SALD structure illustrated the distances between and communication among the amino acids in this network (Fig. 3C). The mapping of the conserved amino acids revealed that NAD^+^ and SAL were surrounded by them. Among those amino acids, Asn149 and Glu250 may bind SAL, while Trp148, Phe226, Gly228, and Phe381 may bind NAD^+^. Thus, we propose here that the conserved and coevolving amino acids in SALD play important roles in catalysis and in substrate and cofactor binding.

### MD simulation

To explore the potential function of the amino acids in SALD, we first constructed a 3D model of SALDan by homology modeling using the Modeller 9 program^28^ based on the crystal structures of SALDpp (PDB ID: 4JZ6) and other aldehyde dehydrogenases (PDB ID: 4FR8, 4O6R, 4NMK, 2O2P, and 3PQA). The overall stereochemical parameters for the modeled proteins were measured using G-factor generated by PROCHECK^29^, which showed that 99.8% of the residues were found in allowed regions of the Ramachandran plot (Supplementary Fig. 2). Moreover, 94.3% of the total amino acids were positioned in the most favored regions of the Ramachandran plot. The model of SALDan was used for the further analysis of substrate binding, the molecular dynamics (MD) simulation, and the calculation of binding free energy.

**Figure 4:**
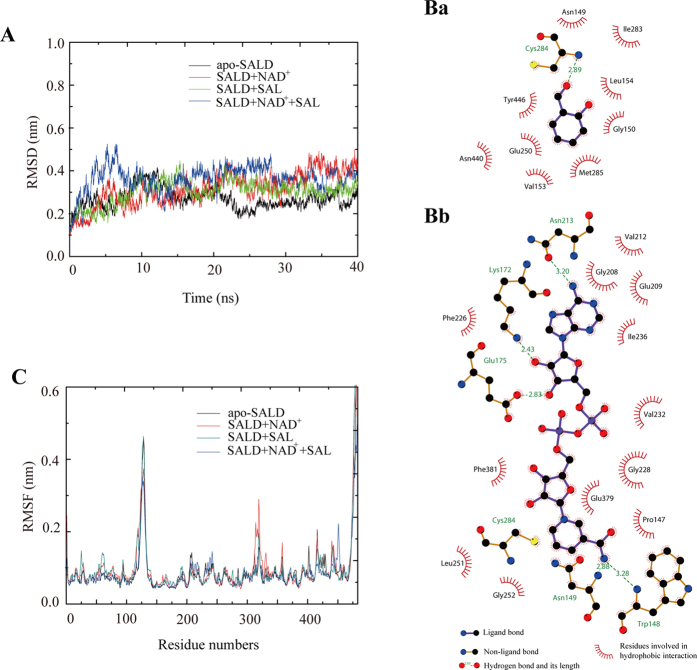
Molecular dynamic analysis of SALDan. (**A**) Backbone RMSDs are shown for SALDan and SALDan complexes at 300 K. Black coloration indicates apo-SALDan; SALDan with NAD^+^ is shown in red; SALDan with SAL is shown in green; and SALDan with both NAD^+^ and SAL is shown in blue. (**B**) The substrate and cofactor binding sites. A two-dimensional representation of ligand–protein interactions for the SALDan–SAL (a) and SALDan–NAD^+^ (b) complexes. (**C**) RMSF of the residue positions of SALDan and SALDan complexes at 300 K. Apo-SALDan, SALDan with NAD^+^, SALDan with SAL, and SALDan with both NAD^+^ and SAL are shown in black, red, blue, and green, respectively.

The complexes of SALD with SAL and/or NAD^+^ were built by superimposing these substrates from the crystal structures of SALDpp complexed with SAL^17^ and human aldehyde dehydrogenase complexed with NAD^+^ on that of SALDan^30^. The complex structure based on the superimposition results was used as the initial structure for MD simulations, which were performed using GROMACS software^31^ to investigate the conformational changes and protein internal motions within a nanosecond timescale for apo-SALDan, SALDan-NAD^+^, SALDan-SAL, and SALDan-NAD^+^-SAL. In the simulation, the root-mean-square deviation (RMSD) is a crucial parameter of convergence in protein structure changes over the course of a simulation. The backbone RMSD of apo-SALDan equilibrated around 0.28 nm after 20 ns of simulation. The backbone RMSDs of SALDan-NAD^+^, SALDan-SALD, and SALDan-NAD^+^-SAL equilibrated around 0.35 nm over the same time frame, as shown in Fig. 4A. SALDan in complex with its substrate and/or cofactor showed a higher RMSD value than the apoenzyme did. This suggests that substrate or cofactor binding causes a conformational change in SALDan. Based on the RMSD analysis, the first 10 ns MD trajectory was deleted and the remaining 30 ns trajectory was used in the production analysis.

The predicted binding mode of SALDan with NAD^+^ based on the MD simulation is illustrated in Fig. 4B. In the MD simulation, the analyses revealed that SALDan can bind to SAL, with the side chains of the Asn149, Gly150, Val153, Leu154, Glu250, Ile283, Met285, Asn440, and Tyr446 residues localized to the active region. The NAD^+^ molecule has numerous interactions with residues in the α/β folds through hydrophobic interaction and hydrogen bonds. Briefly, the adenine dinucleotide part of NAD^+^ is stabilized by Gly208, Glu209, Val212, Phe226, Gly228, Val232, Ile236, Glu379, and Phe381. The dinucleotide also forms a hydrogen bond with Lys172, Glu175, and Asn213. The nicotinamide of NAD^+^ interacts with Trp148 and Asn149 via hydrogen bond formation. Pro147, Leu251, and Gly252 contribute the binding by hydrophobic interactions. Cys284 is positioned close to both NAD^+^ and SAL, implicating it as a potentially important residue.

In contrast to RMSD, which is a global measurement of the protein motion, root-mean square fluctuation (RMSF) can be used to analyze the flexibility of each residue present in the systems (Fig. 4C). The results of RMSF calculations showed that the four systems followed a similar pattern of fluctuation, and the highest RMSF values were calculated for the amino acids at the C-terminus, which suggested that the C-terminus is the most flexible region of SALDan. However, there are differences in the fluctuation patterns of the complexes and apo-protein during 30 ns of simulation. The average RMSF values per residue in the NAD^+^ binding sites for apo-SALDan and the SALDan-NAD^+^ complex were 0.76 and 0.62 Å, respectively. The average RMSF values per residue for the SAL binding sites of apo-SALD and the SALDan-SAL complex were 0.71 and 0.64 Å, respectively. After binding both NAD^+^ and SAL, the average RMSF values per residue in the NAD^+^ binding sites and SAL binding sites were 0.64 and 0.65 Å, respectively. This indicates that these residues become more rigid after binding to the cofactor and/or substrate. We suggested that this decrease in flexibility allows the protein to undergo the proper conformational change for catalysis.

### Free energy analysis for the wild-type complexes

**Figure 5:**
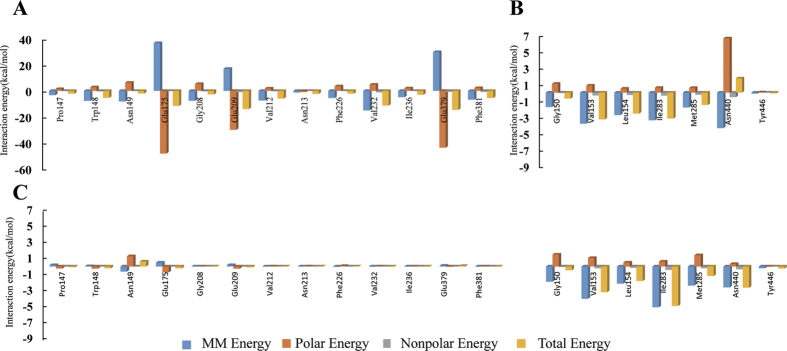
The decomposition of the binding energy on a per-residue basis in the binding sites of the SALDan–NAD^+^ complex (**A**), SALDan–SAL (**B**), and SALDan–NAD^+^-SAL complex (**C**). (Blue histograms: molecular mechanics energy; orange histograms: polar energy; gray histograms: nonpolar energy; yellow histograms: total energy).

Quantification of the average energy of the interaction between NAD^+^/SAL and the specific residues located in the binding sites could provide further insight about which residues were important for the substrates’ binding. Therefore, ligand-residue interaction decomposition was performed by the molecular mechanic/Poisson–Boltzmann surface area (MM/PBSA) method using the g_mmpbsa package^32^,^33^. The summations of the per-residue interaction free energies were separated into molecular mechanics energy (Δ*E*_*MM*_), polar binding energy (Δ*G*_*polar*_), non-polar solvation free energy (Δ*G*_*np*_), and total contribution (Δ*G*_*total*_). The binding amino acids residues listed in Fig. 4B are selected and the energy contributions from those residues are summarized in Fig. 5. As shown, the obtained results for the SALDan-NAD^+^ complex showed that Glu175, Glu209, and Glu379 had an appreciable polar binding energy (Δ*G*_*polar*_) contribution, with Δ*G*_*polar*_ values of −48.21, −29.99, and −43.72 kcal/mol, respectively (Fig. 5). In addition, the residues Trp148, Asn149, Gly208, Val212, Phe226, Val232, and Phe381, which all had Δ*G*_*np*_ values of ≤−6.8 kcal/mol, had strong hydrophobic interactions with the ligand. In the SALDan-SAL complex, Val153, Leu154, Ile283, and Asn440 in the binding cavity had the appreciable Δ*E*_*MM*_ values of −3.79, −2.74, −3.38, and −4.34 kcal/mol, respectively, which indicated that SALD can bind the substrate with the binding cavity effectively. The calculated Δ*G*_*total*_ values for the ligands of the SALDan-NAD^+^ and SALDan-SAL complexes contributed by the selected residues were −82.87 kcal/mol and −9.59 kcal/mol, respectively, which indicated that the protein can bind NAD^+^ much more strongly than SAL. The binding energy of the ligands in the SALDan-NAD^+^-SAL complex was also analyzed. Interestingly, the calculated Δ*G*_*total*_ values for NAD^+^ and SAL in the complex were changed to 0.04 kcal/mol and −14.45 kcal/mol, respectively. The dramatic change of the Δ*G*_*total*_ values with NAD^+^ binding after the ternary complex formation suggested that a conformational change occurred in the NAD^+^ binding site, which may facilitate NADH release. In contrast, the binding to SAL was increased after the formation of the ternary complex. Among the binding amino acids, Ile283 and Asn440 had the largest contributions to the increase, with decreased Δ*G*_*total*_ values of 1.74 kcal/mol and 4.37 kcal/mol, respectively. Based on the above data, it would appear that the amino acids in the active site have varying contributions to ligand binding and a conformational change is induced during the binding process.

### Purification of SALDan

**Figure 6:**
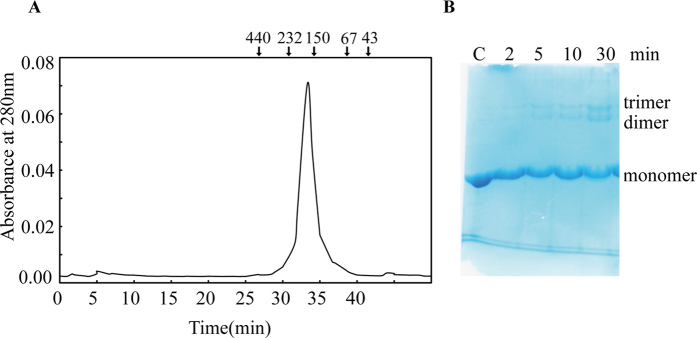
Oligomerization of SALDan. (**A**) Gel filtration chromatography of SALDan. Purified SALDan was analyzed using a Superdex 200 10/300 GL column. The molecular standard proteins are ferritin (440 kDa), catalase (232 kDa), alcohol Dehydrogenase (140 kDa), albumin (67 kDa), and ovalbumin (43 kDa). (**B**) Cross-linking of SALDan with aldehyde. The purified SALDan (0.5 mg/ml) proteins were cross-linked with 0.1% aldehyde for the indicated period at 25 °C. After incubation, the samples were treated with a one-fourth volume of 1 M Tris-HCl and subjected to SDS-PAGE followed by staining with Coomassie blue R-250 as described in the Materials and Methods.

The gene encoding SALDan was cloned and overexpressed in *E. coli* and SALDan was purified using an Ni-NTA column (Supplementary Fig. 3). The molecular mass of purified SALDan was approximately 53 kDa. To examine the native structure of SALDan, the purified protein was analyzed by gel filtration chromatography (Fig. 6A) and protein cross-linking (Fig. 6B). One peak was eluted from the gel filtration column at approximately 160 kDa. To determine whether SALDan can form a stable complex under *in vitro* conditions, cross-linking analysis was performed by incubating SALDan with aldehyde for various periods. The cross-linked products were then separated by SDS-PAGE followed by Coomassie blue staining. The results showed that the subunits of the SALDan proteins are cross-linked to various intermediates corresponding to sizes from monomers to trimers. Upon prolonged incubation, no additional band appeared. These results suggest that SALDan consists of three subunits in its native form.

### Kinetics of SALDan

The influences of pH and temperature on the activity of purified SALDan were examined. The specific activity of purified SALDan toward SAL was examined in the pH range 4.5–9.0 using a mixture of different buffers including sodium acetate, HEPES, glycine, and sodium phosphate (Supplementary Fig. 4A). The optimum pH for enzyme activity was approximately 7.5, which is well within the pH range (pH 6.0–9.0) that is suitable for the growth of *A. naphthalenivorans*^34^. The optimum pH of SALDan was different from that of the SALD of *Pseudomonas* spp. C6 and G7 (optimal pH 8.0–8.5), which reflects the differences in the environmental conditions of these organisms. SALDan activity was measured at temperatures from 15–45 °C (Supplementary Fig. 4B) and exhibited high activity from 25–35 °C. SALDan catalyzes a two-substrate reaction. The *K*_m_ values for NAD^+^ and SAL were determined by varying the concentration of one substrate (SAL or NAD^+^) in the presence of a constant concentration of the other substrate. The plot of velocity versus [NAD^+^] substrate showed a typical Michaelis–Menten profile. The *K*_m_ and *V*_max_ values for NAD^+^ were 39.5 ± 3.2 μM and 94.1 ± 11.5 U/mg, respectively (Table 1). A direct plot of velocity versus [SALD] was not observed to be hyperbolic in nature (as for linear inhibition), but had an unusual shape. The velocity increased together with concentration elevation and the highest velocity could be achieved at 40 μM. The activity was approximately 80% of the maximal velocity when assayed at 200 μM, indicating the characteristic of substrate inhibition. The *K*_m_ and *V*_max_ for SAL were calculated to be 3.8 ± 0.5 μM and 49.5 ± 4.5 U/mg, respectively, and the *K*_i_ of SALDan was 378.7 ± 45.2 μM (Table 1).

The substrate specificity of SALDan was investigated using a variety of aldehydes (Table 1, Supplementary Fig. 5). SALDan oxidizes wide range of aldehydes, especially aromatic aldehydes (SAL, benzaldehyde, chlorobenzaldehyde, nitrobenzaldehyde, and naphthaldehyde). The apparent *K*_m_ values for aromatic aldehydes ranged from 2.3–6.5 μM. *V*_max_ values for aromatic aldehydes also showed a narrow range from 22.6–74.2 U/mg. Finally, the catalytic efficiencies (App *k*_cat_/*K*_m_ (s^−1^ _•_ μM^−1^) of these substrates were all within the same order of magnitude. SALDan catalyzes the oxidation of linear aliphatic aldehydes with short chains with *k*_cat_ values from 30.4–54.0 s^−1^. However, SALDan exhibited *K*_m_ values toward short-chain aliphatic aldehydes that were much higher than those toward aromatic substrates, which caused short-chain aliphatic aldehydes to have *k*_cat_/*K*_m_ values incomparable with those of aromatic aldehydes.

### Site-directed mutagenesis of SALDan

The amino acids evolution and MD simulation analyses revealed that Asn149 is highly conserved and involved in both NAD^+^ and SAL binding. Additional free energy analysis further demonstrated that Val153 and Glu175 contributed significant free energy to binding NAD^+^ and SAL, respectively. These three amino acids were each mutated to alanine to study their functions (Supplementary Fig. 2). The catalytic efficiency of N149A for SAL decreased by about three times compared with that of the wild-type protein (Table 2, Supplementary Fig. 6). In contrast, V153A had no significant effect on the kinetic parameters for either NAD^+^ or SAL. Finally, the mutation of Glu to Ala at position 175 had a remarkable effect on the catalytic activity. The apparent *K*_m_ value of E175A to NAD^+^ increased by about two times and the *k*_cat_/*K*_m_ toward the cofactor decreased by four times. The *K*_m_ value of the mutant toward SAL also increased and the catalytic efficiency decreased by six times. These site-directed mutagenesis experiments demonstrated that the mutation of the selected three amino acids affected enzyme activity to some extent, but not decisively, compared to that of the wild-type enzyme.

## Discussion

In this study, we first performed a large-scale *in silico* analysis of SAL dehydrogenases (SALDs), which revealed that SALDs are distributed among both bacteria and fungi. The evolutionary relationship of these enzymes was established by the SSN and phylogenetic tree analyses. Evolutionary studies indicated that the residues that are directly involved in substrate/cofactor binding and catalytic activity are highly conserved and have coevolved. Furthermore, the amino acids in SALD from *A. naphthalenivorans* (SALDan) that contributed to binding the substrate/cofactor were identified by MD simulation and free energy analysis. Finally, we purified and characterized SALDan, which was found to be capable of oxidizing aromatic aldehydes and had broad substrate specificity. Point mutations of the amino acids binding the substrate/cofactor had no detrimental effect on the enzyme activity.

SALD and the homologues were particularly abundant in bacteria (81.65%), followed by fungi (18.35%) (Fig. 1). This may be because the majority of PAH-degrading microorganisms are bacteria and their metabolic mechanisms and pathways have been well-studied^35^. In fungi, the phylum *Ascomycota*, the subphylum *Mucoromycotina*, and the phylum *Basidiomycota* are the main contributors to PAH degradation in polluted environments^36^. Our studies also showed that the proteins showing high similarity to SALD existed in the phyla *Ascomycota* and *Basidiomycota* of the fungal kingdom. The detailed pathway of PAH degradation in fungi is not clear and several possible degradation pathways have been suggested^37^,^38^. In the PAH degradation process of fungi, fungal enzymes such as lignin peroxidase, laccase, cytochrome P450 monooxygenase, epoxide hydrolases, lipases, protease, and dioxygenases may be involved in oxidizing the PAH compounds^37^. To our knowledge, the fungal proteins showing high similarity to SALD in bacteria have not been reported or reviewed by the Swiss-Prot database (Fig. 1). Considering that the fungal strains harboring the enzymes may be capable of degrading PAH, we speculate that the SALD-homologous proteins in fungi may also play a role in aromatic hydrocarbon degradation.

The aldehyde dehydrogenase family members contain two conserved sites: a cysteine active site and a glutamic acid active site^39^. The cysteine residue acts as a nucleophile and attacks the carbonyl carbon of the aldehyde to form an intermediate^40^, while the glutamic acid residue acts as a general base in the hydrolysis of the acyl-enzyme intermediate^41^. In the evolutionary analysis of SALD, one cysteine residue was found to be highly conserved, while the Glu residue corresponding the active site was not as conserved as the cysteine (Fig. 2). This may be because the enzymes have very distinct catalytic properties, even though their subunit structures are similar. Further experiments also showed that site-directed mutagenesis of the conserved glutamic acid residues 209 and 333 to glutamine residues in class 3 human aldehyde dehydrogenase had only marginal effects on enzyme activity^42^. In addition to the catalytic residues, five residues (Pro147, Trp148, Asn149, Gly228, and Phe381) that bind NAD^+^ and one residue (Asn149) that binds SAL were also found to be highly conserved through MD simulation using SALDan as the example (Fig. 2). The ordering of conservation was determined as catalytic residues >NAD^+^ -binding residues >SALD-binding residues. In SALDan, Asn149 stabilizes both NAD^+^ and SAL among these amino acid residues. Mutation of Asn149 caused the catalytic efficiency to both substrates to decrease by 50% (Table 2). Meanwhile, mutation of Glu175, which provides the lowest free energy for binding NAD^+^, increased *K*_m_ to NAD^+^ by 5-fold. However, the mutation of Val153, which exhibited the lowest free energy for binding SAL, had a marginal effect on the catalytic parameters. This may be because Val to Ala replacement does not affect the positioning of the amino acids. On the other hand, because SALD and its homologous proteins always show a broad substrate specificity and have a wide pocket to bind the aromatic ring aldehydes, the mutation of one residue will not affect the enzyme activity dramatically.

**Figure 7:**
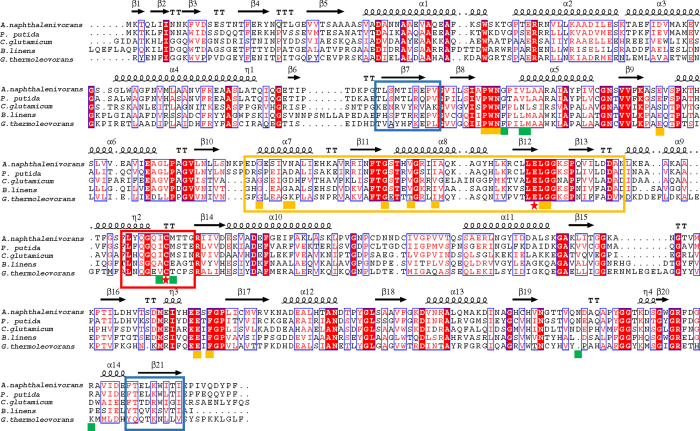
Sequence alignments of aldehyde dehydrogenases towards the aromatic aldehydes with broad specificity. Identical residues are shown in white letters with a red background, similar residues are shown in red letters with a white background, and varied residues are shown in black letters. The predicted secondary structure is shown at the top of the alignment. α-Helices are represented as helices and β-strands as arrows, while β-turns are identified by ‘TT’ and 310-helices by η. The core regions of the catalytic domain, NAD^+^ binding domain, and oligomerization domain are boxed by red, orange, and green rectangles, respectively. Amino acid residues involved in catalysis, NAD^+^ binding, and SAL binding are labeled by a red star, orange square, and green square, respectively. Aldehyde dehydrogenases are from *A. naphthalenivorans* (F5Z5S7), *P. putida* (Q1XGL7), *C. glutamicum* (Q8NMB0), *B. linens* (A0A142NN86), and *G. thermoleovorans* (A0A098L0N3).

The aldehyde dehydrogenases exhibit diverse oligomeric states, including dimer, trimer, tetramer, and hexamer^43^. The aldehyde dehydrogenase from *Corynebacterium glutamicum* exists in dimeric, trimeric, and tetrameric forms, as revealed by gel filtration chromatography^44^. In the case of SAL dehydrogenase, SALDpp generated a dimeric biological unit, as shown by the determination of its crystal structure^45^. The enzyme from *Pseudomonas* sp. C6, which shares 67% sequence identity with SALDpp, has a trimeric form based on gel filtration chromatography analysis^16^. SALDan was also determined to be trimeric based on gel filtration chromatography and protein cross-linking analyses. Despite having different modes of oligomerization, each monomer can be divided into three domains: a cofactor binding domain composed of a core that resembles the Rossmann fold, a catalytic α/β domain, and a small protruding domain that enables oligomerization^45^,^46^. The oligomerization domains of SALD are located in the β7, β21, and C-terminus regions (Fig. 7). RMSF analysis showed that the highest amount of fluctuation was present in the C-terminus region, which suggested that the oligomerization domain is highly flexible. Besides, this domain is less evolutionarily conserved (Figs 2 and 7). Therefore, we speculate that the diversity of the oligomeric forms of the aldehyde dehydrogenases is caused by both the instability in structure and the low evolutionary conservation of the oligomerization domain.

The substrate specificities of the aldehyde dehydrogenases are determined by their catalytic domains. SALDan, SALDpp, and SALD from *Pseudomonas* sp. C6 all show activity towards a broad range of aromatic aldehyde substrates. The aldehyde dehydrogenase from *Corynebacterium glutamicum*, which has three oligomeric forms, catalyzes the oxidization of *p*-hydroxybenzaldehyde, 3,4-dihydroxybenzaldehyde, *o*-phthaldialdehyde, cinnamaldehyde, syringaldehyde, and benzaldehyde^44^. The same properties have been reported for the aldehyde dehydrogenases from *Geobacillus thermodenitrificans*^47^ and *Brevibacterium* sp. KU1309^48^. Sequence alignments of the enzymes that have a broad substrate specificity showed that the catalytic residues are highly conserved, and the cofactor binding residues and core region of the cofactor binding domain are also well conserved. However, the amino acids that bind the aromatic aldehydes are less conserved than other regions, although all of the amino acids fulfilling this function were hydrophobic (Fig. 7). The property of a low evolutionary conservation can be extended to all SALDs (Fig. 2). The structure of SALDpp highlighted that the dimensions of the substrate binding pocket and its hydrophobic environment allow a broad substrate spectrum. Here, we propose that the evolutionary flexibility of the amino acids in the substrate binding pocket also contributes to the wide variety of substrates of the SALD subfamily.

In conclusion, our results highlight that SALD and its homologous proteins exist in both bacteria and fungi. The key residues for catalysis and cofactor binding have been conserved during evolution, but the residues binding the substrate are less well conserved. The domain for oligomerization with a flexible structure is also less well conserved. Finally, we characterized SALDan as having a broad range of substrates, which may be related to the evolutionary flexibility of the amino acids in the substrate binding pocket.

## Materials and Methods

### Collection of SALD and construction of SSN

A protein BLAST search was performed using SALDan as a query sequence in the UniProt or env_nr database with a cut-off e–value of 10^−80^ (>40% sequence identity)^20^. The proteins identified as homologous from the two databases are listed in Supplementary Dataset 1 and 2, respectively. An SSN of the homologous proteins obtained via BLAST was constructed using the Enzyme Function Initiative-Enzyme Similarity Tool^22^ and visualized by Cytoscape 3.3^49^. Each node in the network indicates a protein and the edge indicates that the two nodes share significant similarity with an e-value less than the selected cutoff.

### MSA and coevolving protein residues

MSA of protein sequences were performed using the ClustalW (version 2) software program^50^. The phylogenetic trees were constructed with MEGA7 using the neighbor-joining method and a bootstrap test was carried out with 1000 iterations^51^,^52^. Analysis of coevolving residues was carried out by calculating MI between two positions in the MSA. MI reflects the extent to which knowing the amino acid at one position can predict the amino acid identity at another position. MI was calculated between pairs of columns in the MSA using the MISTIC approach and web server^23^.

### MD simulation and binding free energy calculation

The three-dimensional structure of SALDan was modeled using the Modeller 9 software program^28^. Structural validation of the enzyme was performed by creating a Ramachandran plot using the PROCHECK server^29^. SALDan-SAL complex was built by superimposing the crystal structure of SALDpp (PDB ID: 4JZ6) on that of SALDan, then deleting the protein structure in 4JZ6. SALDan-NAD^+^ was built by superimposing the crystal structure of human aldehyde dehydrogenase (PDB ID: 4FR8) on that of SALDan, then deleting the protein structure in 4FR8. The SALDan-NAD^+^-SAL was built by the same approach by superimposing the structure of SALDan-NAD^+^ on that of SALDan-SAL^53^,^54^. The structure was used as a starting geometry and parameters for NAD^+^ and SAL were generated with the ACPYPE tool using the general AMBER force field^55^ with AM1-BCC atomic charges. Each system was solvated in a cubic box full of explicit TIP3P water molecules with a 10 Å buffer along each dimension. The systems were neutralized by adding explicit counter ions (Na^+^-Cl^−^) for each complex system. An MD simulation was performed using the GROMACS 5.0.4 software program with the AMBER force field^56^ implemented on a LINUX operating system. To ensure that the system was relaxed and has no serious clashes or unsuitable geometry, the potential energy of the system first needed to be minimized by the steepest descent energy minimization method. The system was then gently heated by incrementing the temperature from 0 to 300 K at constant volume and using periodic boundary conditions. A velocity-rescaling^57^ thermostat was used for temperature coupling with a constant number of atoms, volume, and temperature run for a duration of 200 ps using an ensemble with a constant temperature of 300 K and a coupling constant of 0.1 ps. A constant number of atoms, pressure, and temperature run were performed for 400 ps using an ensemble with a constant pressure of 1 bar and a coupling constant of 0.1 ps. The isotropic Berendsen protocol was used for pressure coupling. Long-range electrostatic effects were modeled using the particle-mesh-Ewald method^58^. A 12 Å cutoff was applied to Lennard–Jones and electrostatic interactions. Bond lengths involving bonds to hydrogen atoms were constrained using the LINCS algorithm^59^. Afterwards, an equilibration production MD simulation was run for 40 ns at constant temperature and pressure. The gmx rmsd and gmx rmsf programs in the GROMACS 5.0.4 was used to obtain the RMSD and RMSF, respectively^31^.

### Calculation of binding free energy

Two hundred and fifty snapshots were extracted from the last 25 ns along the MD trajectory at an interval of 100 ps. The MM/PBSA method was performed using the g_mmpbsa package^32^,^33^ to calculate the binding free energy of the enzyme and substrate. The MM/PBSA method can be conceptually summarized as three energetic terms:


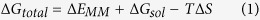


where Δ*G*_*total*_ denotes the binding free energy, Δ*E*_*MM*_ denotes the difference in molecular mechanics energy between the complex and each binding partner in a vacuum, Δ*G*_*sol*_ denotes the solvation free energy, and TΔ*S* denotes the entropy change. Δ*E*_*MM*_ can be further divided into two parts:


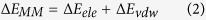


where Δ*E*_*ele*_ and Δ*E*_*vdw*_ denote the electrostatic interaction and van der Waals energy in a vacuum, respectively. In addition, the solvation free energy can also be divided into two parts:


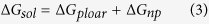


where Δ*G*_*polar*_ and Δ*G*_*np*_ denote the polar and non-polar solvation free energies, respectively. For Δ*G*_*polar*_, the dielectric constants of the solute and solvent were set to 2.0 and 80.0 in our calculations. For Δ*G*_*np*_, the values of coefficients γ and β were set to 0.0054 kcal/mol/A^2^ and 0.92 kcal/mol, respectively.

The entropy change (TΔ*S*) arises from changes in the translational, rotational, and vibrational degrees of freedom. The calculation of entropy change is extremely time-consuming and inaccurate, and for similar protein-inhibitor complex systems the entropy change is similar^60^. Therefore, in our study, we ignored the calculation of entropy change.

### Cloning and site-directed mutagenesis of SALDan

PCR using *A. naphthalenivorans* genomic DNA as a template was performed to isolate *SALDan* using the following oligonucleotide primers: forward: 5′-CG GGATCC ATG AAT AAT CAA GAA CTC TTA AGC-3′ and reverse: 5′-CCC AAGCTT TCA TAT CGGA TAT ATC AGAG AGT T-3′ (the underlined bases indicate the restriction enzyme sites for *Bam*HI and *Hind*III). The PCR product and the pET28-(a) vector were digested by those two restriction enzymes. The ligation products were transformed into *Escherichia coli* BL21 (DE3) cells by electroporation and confirmed by sequencing.

The primers used for the single amino acid mutant were as follows: N149A, forward primer, 5′-ATA GCA CCG TGG GCC GGG CCT ATT GTA TTA-3′; reverse primer, 5′-TAA TAC AAT AGG CCC GGC CCA CGG TGC TAT-3′; V153A, forward primer, 5′-AAT GGG CCT ATT GTA TTA GCG GCT CGG-3′; reverse primer, 5′-CCG AGC CGC TAA TAC AAT AGG CCC ATT-3′; E175A, forward primer, 5′-TTTAAAGCTTCAGAAGTAAGTCCTAAA-3′, reverse primer, 5′-GTT GGG ACA GTC TTC CAG CCC-3′; E175A, forward primer, 5′-ATG CTC CAC CAA ATT GGG CAC-3′; reverse primer, 5′-TTT AGG ACT TAC TTC TGA AGC TTT AAA-3′. The PCR was performed using *Pfu* polymerase, and the cycling parameters were: 95 °C for 5 min (one cycle), 95 °C for 30 s, and 68 °C for 12 min (12 cycles). After amplification, the PCR mixture was digested with *Dpn*I and then transformed into *E. coli* BL21(DE3) by electroporation^61^. The mutants were confirmed by DNA sequencing.

### Expression and purification of SALDan

*E. coli* BL21(DE3) cells containing the pET28a-*SALDan* plasmid were cultured in 2 L of LB broth containing kanamycin (30 μg.mL^−1^) at 37 °C for 3 h. When the OD_600_ reached 0.7, isopropyl-β-d-thiogalactopyranoside was added to a final concentration of 1 mM to induce protein expression. After 4 h of culture with shaking, cells were harvested by centrifugation for 10 min at 4 °C^62^. The cell pellets were resuspended in lysis buffer containing 50 mM Tris-HCl (pH 8.0), 300 mM NaCl, 20 mM 2-mercaptoethanol, and 20 mM imidazole. The cell suspension was sonicated and the supernatants were collected following centrifugation and loaded on a Ni-NTA column. After washing the column with lysis buffer, SALDan was eluted using an imidazole gradient (50–250 mM). The purified SALDan was visualized after separation by 12% sodium dodecyl sulfate polyacrylamide gel electrophoresis (SDS-PAGE). After dialysis with 50 mM HEPES buffer (pH 8.0) containing 150 mM NaCl and 20 mM 2-mercaptoethanol to remove metal ions, the purified proteins were stored at −80 °C. Protein concentrations were estimated by the method of Bradford using bovine serum albumin as a standard^63^.

### Effects of temperature and pH on SALDan activity

The activity of the purified proteins was determined using SAL as a substrate. A reaction volume of 500 μL was prepared in a microfuge tube containing purified SALDan (0.1 μM), SAL (100 μM), and NAD^+^ (100 μM) dissolved in 50 mM HEPES (pH 7.5) containing 150 mM NaCl. The SALDan activity was monitored as the rate of appearance of NADH at 340 nm spectrophotometrically. The specific activity of the enzyme (units.mg^−1^.min^−1^) was expressed as the amount of enzyme required to produce 1 μM of NADH under the assay conditions. The influence of pH on the activity was determined using the protocol described above, with the exception of replacing the Tris-HCl buffer with 50 mM sodium acetate (pH 3.0–5.0), 50 mM 2-(*N*-morpholino)ethanesulfonic acid (pH 5.0–7.5), 50 mM HEPES (pH 8.0–8.5), 50 mM glycine (pH 9.0–10.0), or 50 mM sodium phosphate (pH 11.0)^52^. All assays were performed at the optimal temperature. To determine the influence of temperature on the enzymatic activity, the reactions were performed at 15, 20, 25, 30, 35, 40, and 45 °C, respectively.

### Kinetics assay of SALDan

For kinetic studies of aldehydes, the initial velocities of the enzymatic reactions were examined by varying the concentrations of various aromatic as well as aliphatic aldehydes in the presence of NAD^+^ (100 μM) and the enzyme (0.1 μM) under optimal conditions. For substrates that absorb at 340 nm, thus interfering with the calculation of the initial rate, their molar coefficients (ε) were calculated as described previously^64^ and are presented in Table S1. The apparent kinetic parameters were calculated using the equation:


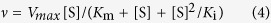


The apparent kinetic parameters for NAD^+^ were determined at a fixed concentration of SAL (50 μM) and a varying concentration of NAD^+^ (0–500 μM). The apparent *K*_m_ and *V*_*max*_ values were calculated by using the equation:


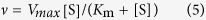


All the activity data were determined by three separate experiments with at least three technical replicates.

## Additional Information

**How to cite this article**: Jia, B. *et al*. Evolutionary, computational, and biochemical studies of the salicylaldehyde dehydrogenases in the naphthalene degradation pathway. *Sci. Rep.* **7**, 43489; doi: 10.1038/srep43489 (2017).

**Publisher's note:** Springer Nature remains neutral with regard to jurisdictional claims in published maps and institutional affiliations.

## Supplementary Material

Supplementary Table and Figures

Supplementary Dataset 1 and 2

## Figures and Tables

**Table 1:** Substrate specificity of the recombinant SALDan.

Substrates	App *K*_m_ (μM)	App *V*_max_ (U/mg)	App *k*_cat_ (s^−1^)	App *k*_cat_/*K*_m_ (s^−1^ _•_ μM^−1^)	App *K*_i_ (μM)
Salicylaldehyde	3.8 ± 0.5	49.5 ± 4.5	123.6 ± 13.6	32.5 ± 4.2	378.7 ± 45.2
Benzaldehyde	2.3 ± 0.3	22.6 ± 3.8	56.1 ± 10.5	24.2 ± 2.9	562.6 ± 87.6
2-Chlorobenzaldehyde	3.2 ± 0.3	45.6 ± 6.2	114.2 ± 17.2	35.7 ± 4.5	356.4 ± 40.1
3-Chlorobenzaldehyde	4.8 ± 0.7	56.8 ± 7.2	142.1 ± 20.5	29.6 ± 3.5	123.9 ± 20.1
4-Chlorobenzaldehyde	5.5 ± 1.1	74.2 ± 5.8	185.5 ± 16.1	33.7 ± 2.8	132.1 ± 18.9
4-Nitrobenzaldehyde	6.5 ± 0.9	32.3 ± 4.6	80.2 ± 14.5	12.3 ± 2.0	265.7 ± 35.8
2-Naphthaldehyde	3.4 ± 0.5	61.4 ± 5.1	153.1 ± 14.4	45.0 ± 4.4	248.5 ± 22.5
Formaldehyde	12360 ± 1820.0	3.3 ± 0.7	8.6 ± 2.2	7.0E−04 ± 8.4E−03	—
Butyraldehyde	3200 ± 235.0	5.9 ± 1.0	14.3 ± 2.8	4.5E−03 ± 5.7E−02	—
Glutaraldehyde	560 ± 46.0	11.6 ± 1.2	29.6 ± 3.7	5.3E−02 ± 4.9E−01	—

“—” means not observed.

**Table 2:** Apparent kinetic parameters of the wild-type and mutant SALDan enzymes.

Proteins	Substrates	App *K*_m_ (μM)	App *V*_max_ (U/mg)	App *k*_cat_ (s^−1^)	App *k*_cat_/*K*_m_ (s^−1^ _•_ μM^−1^)	App *K*_i_ (μM)
WT	NAD^+^	39.5 ± 3.2	94.1 ± 11.5	234.3 ± 24.2	5.9 ± 0.5	—
	SALD	3.8 ± 0.5	49.5 ± 4.5	123.6 ± 13.6	32.5 ± 4.2	378.7 ± 45.2
N149A	NAD^+^	52.3 ± 6.1	83.5 ± 10.8	208.8 ± 25.6	4.0 ± 0.8	—
	SALD	9.5 ± 1.3	38.6 ± 3.5	100.4 ± 16.8	10.6 ± 1.3	446.7 ± 52.6
V153A	NAD^+^	44.9 ± 4.2	102.6 ± 9.8	254.5 ± 21.5	5.7 ± 0.5	—
	SALD	4.6 ± 0.7	59.5 ± 7.6	153.5 ± 20.4	33.4 ± 4.7	428.3 ± 49.4
E175A	NAD^+^	105.6 ± 10.3	60.6 ± 6.9	157.6 ± 17.2	1.5 ± 0.4	—
	SALD	15.1 ± 2.6	32.3 ± 4.3	82.2 ± 10.1	5.4 ± 0.9	461.6 ± 51.4

“—” means not observed.
