# Supplementary material for: Evolutionary, computational, and biochemical studies of the salicylaldehyde dehydrogenases in the naphthalene degradation pathway
Source: Sci Rep. 2017 Feb 24;7:43489. doi: 10.1038/srep43489 (PMC5324060; doi:10.1038/srep43489)
Supplement: Supplementary Table and Figures [file srep43489-s1.pdf]

Supplementary Information

Evolutionary, computational, and biochemical studies of the salicylaldehyde dehydrogenases in the naphthalene degradation pathway

Baolei Jia[1,2], Xiaomeng Jia[2], Kyung Hyun Kim[2], Zhong Ji Pu[3], Myung-Suk Kang[4], and Che Ok Jeon[2]

[1]School of Bioengineering, Qilu University of Technology, Jinan 250353, China

[2]Department of Life Science, Chung-Ang University, Seoul 06974, Republic of Korea

[3]School of Life Science and Biotechnology, Dalian University of Technology, Dalian 116024, China

[4]Microorganism Resources Division, National Institute of Biological Resources, Incheon 22689, Republic of Korea

Running title: A comprehensive analysis of salicylaldehyde dehydrogenases

Correspondence and requests for materials should be addressed to B.J. (jiabaolei@hotmail.com, baoleijia@cau.ac.kr) and C.O.J. (cojeon@cau.ac.kr).

Supplementary Table 1. Molar absorption coefficient (ε) at 340 nm of aromatic aldehyde and acid and in 50 mM HEPES pH 7.5, and 150 mM NaCl.

Substrate	ε, M^{-1} cm^{-1}	Product	ε, M^{-1} cm^{-1}	Correction factor
Salicylaldehyde	2530	Salicylic acid	52	1.67
Benzaldehyde	51	Benzyl acid	38	1.00
2-Chlorobenzaldehyde	63	2-Chlorobenzoic acid	59	1.00
3-Chlorobenzaldehyde	74	3-Chlorobenzoic acid	63	1.00
4-Chlorobenzaldehyde	88	4-Chlorobenzoic acid	76	1.00
4-Nitrobenzaldehyde	682	4-Nitrobenzyl acid	654	1.00
2-Naphthaldehyde	3542	2-Naphthoic acid	352	2.13

The correction factor was calculated by the equation:
correction factor=ε(NADH)/(ε(NADH)- ε(substrate)+ ε(product)), where ε(NADH)=6200 M^{-1} cm^{-1}.

Supplementary Figure 1

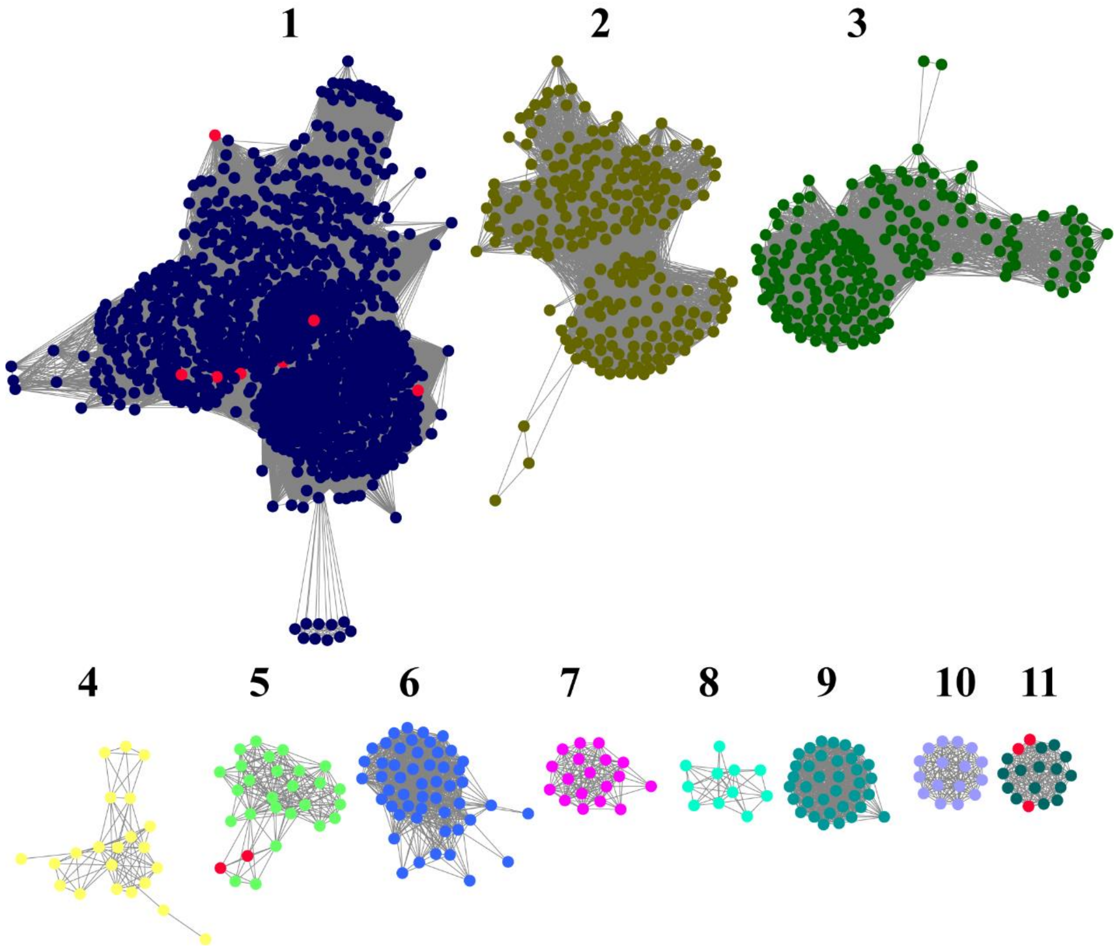

Supplementary Figure 1. Protein sequence similarity network of SALDs from Uniprot and NCBI metagenomics protein database. The network was generated using an e-value of 10^{-150} (> 60% sequence identity). The proteins from Uniprot database were classified into 11 clusters and each cluster was assigned a different color. Proteins from NCBI metagenomics protein database grouped into cluster 1, 5, and 11 were painted by red color. The accession number of proteins from NCBI metagenomics protein database were listed in Supplementary Dataset 2.

Supplementary Figure 2

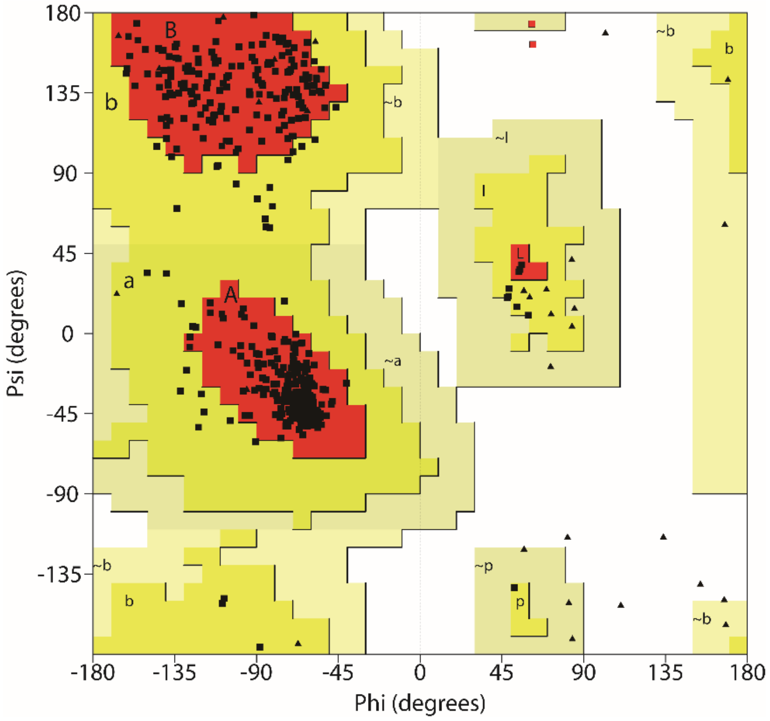

Supplementary Figure 2. Validation of protein structure of SALDan using Ramchandran plot. The Ramachandran plot revealed that 90.3% of amino acid residues from modeled structure were incorporated in the favored regions (A, B, and L) of the plot. 9.2% of the residues were in allowed regions (a, b, l, and p) of the plot.

Supplementary Figure 3

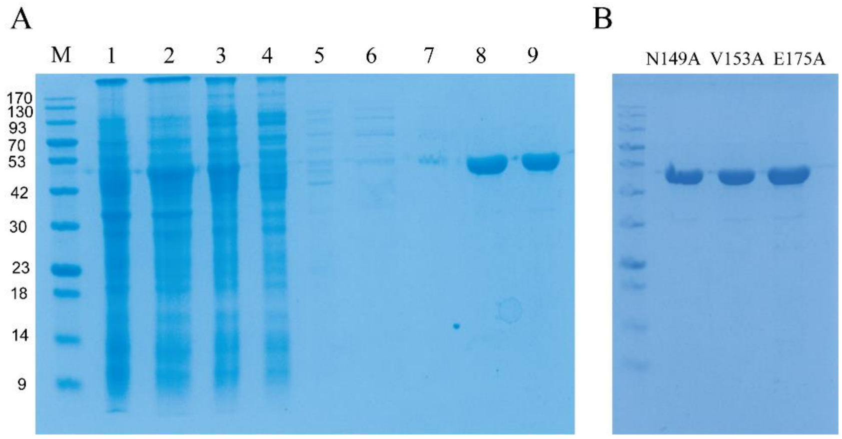

Supplementary Figure 3. Expression and purification of SALDan and the mutants from *Escherichia coli*. A Purification of SALDan, Lane M, protein marker; the molecular mass standards are indicated at the left. Lane 1, crude protein extract from non-induced cells; Lane 2, crude protein extract from IPTG-induced cells; Lane 3, soluble extract from IPTG-induced cells; Lane 4, unbound proteins eluted from the Ni-NTA column; Lane 5-7, proteins eluted with 20 mM, 50 Mm, and 100 mM imidazole, respectively; Lane 8, proteins eluted with 300 mM imidazole. B Purification of the mutant of N149A V153A, and E175A.

Supplementary Figure 4

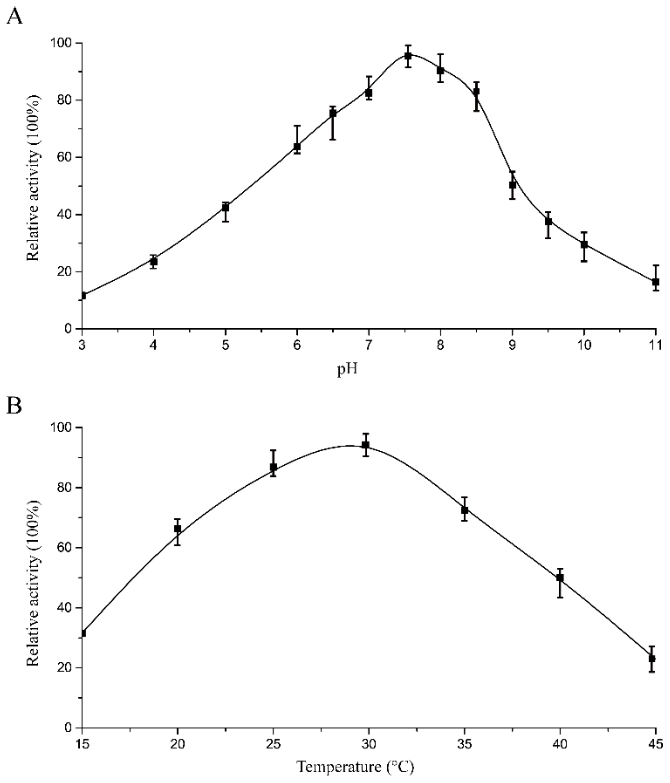

Supplementary Figure 4. A Optimal pH of SALDan. Different buffers were used for the different pH solutions in this assay. Sodium acetate buffer was used for pH 3.0–5.0; MES buffer was used for pH 5.0–7.5; HEPES buffer was used for pH 8 and 8.5; glycine buffer was used for pH 9.0 and 10.0; and Na_2HPO_4 buffer was used for pH 11.0. Values obtained at pH 7.5 were set as 100%. B Optimal temperature of SALDan. Values obtained at 30°C were set as 100%.

Supplementary Figure 5

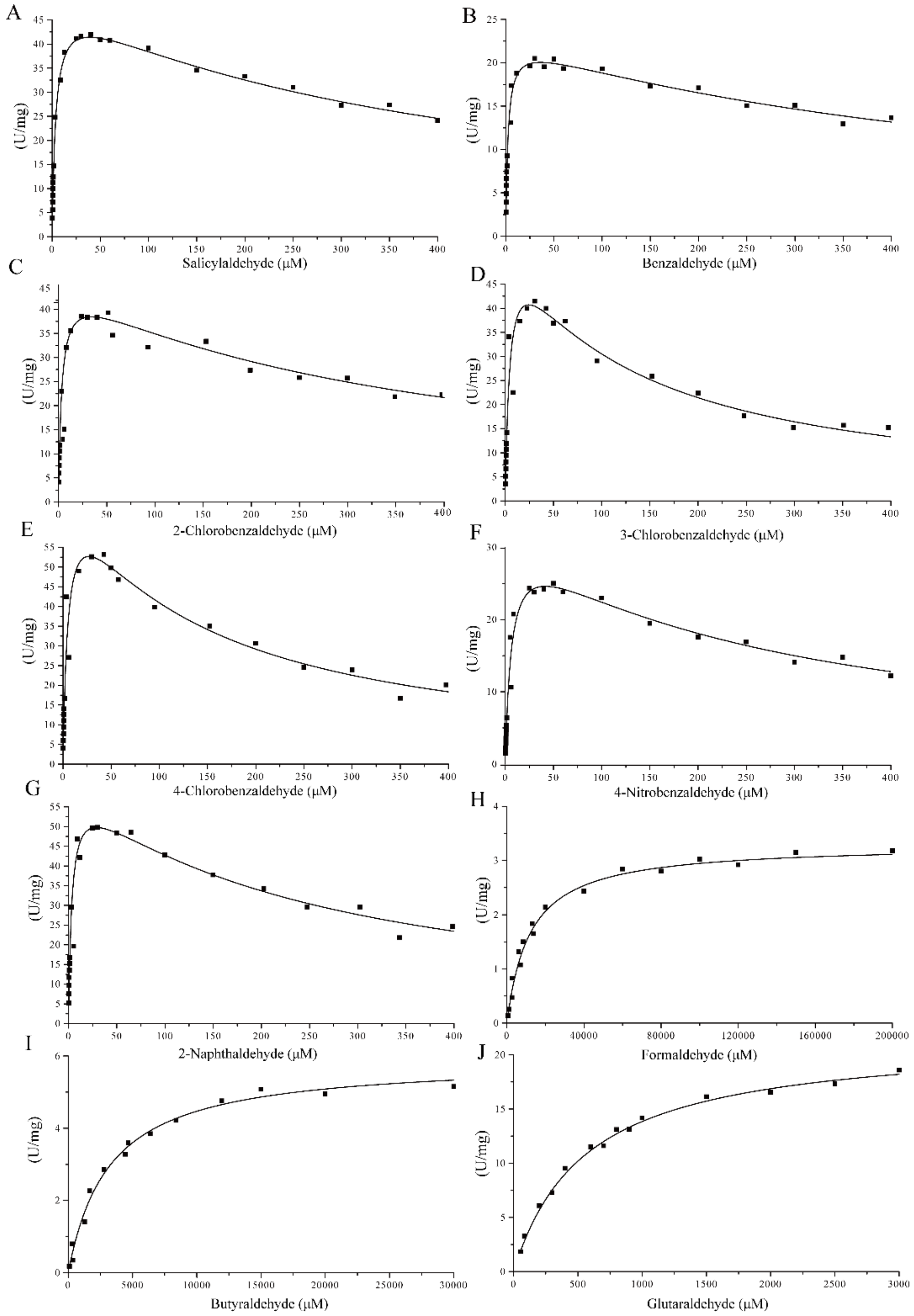

Supplementary Figure 5. Effects of substrates (names shown under X-axis) concentration on the velocity of the SALDan. Assays were performed as described in Section “Materials and Methods.”

Supplementary Figure 6

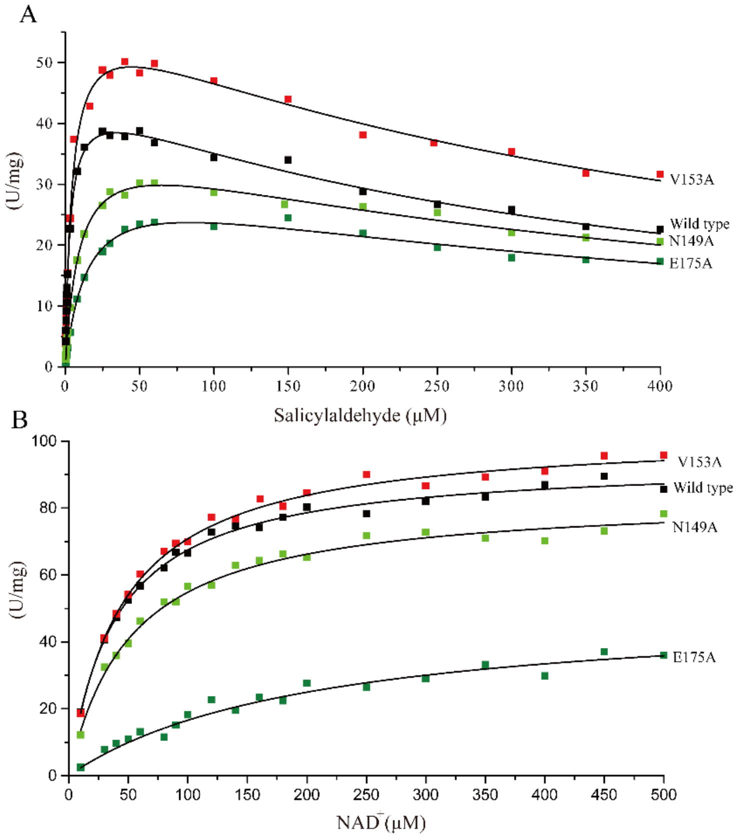

Supplementary Figure 6. Kinetics characteristics of wild-type SALDan (black), V153A (red), N149A (green), and E175A (blue) with salicylaldehyde (A) and NAD^+ (B) as substrates.
